# A Proof-of-Concept Analysis of Plasma-Derived Exosomal microRNAs in Interstitial Pulmonary Fibrosis Secondary to Antisynthetase Syndrome

**DOI:** 10.3390/ijms232314579

**Published:** 2022-11-23

**Authors:** Sara Bozzini, Giovanni Zanframundo, Cecilia Bagnera, Eleonora Bozza, Sara Lettieri, Valentina Vertui, Veronica Codullo, Francesca Cuzzocrea, Belén Atienza-Mateo, Sara Remuzgo Martinez, Carlomaurizio Montecucco, Miguel A. González-Gay, Lorenzo Cavagna, Federica Meloni

**Affiliations:** 1 Research Laboratory of Respiratory Disease, Section of Cell Biology, UOS Transplant Center, IRCCS Foundation Policlinico San Matteo, 27100 Pavia, Italy; 2 Department of Internal Medicine and Therapeutics, University of Pavia, 27100 Pavia, Italy; 3 Division of Rheumatology, IRCCS Foundation Policlinico San Matteo, 27100 Pavia, Italy; 4 Pneumology Unit, IRCCS Policlinico San Matteo Foundation, 27100 Pavia, Italy; 5 Division of Rheumatology and Epidemioogy, Genetics and Atherosclerosis Research Group on Systemic Inflammatory Disease, Hospital Universitario Marqués de Valdecilla, IDIVAL, 39008 Santander, Spain; 6 Department of Medicine and Psychiatry, Universidad de Cantabria, 39005 Santander, Spain

**Keywords:** microRNAs, antisynthetase syndrome, interstitial lung disease

## Abstract

Antisynthetase syndrome (ASSD) is an autoimmune disease characterized by the positivity of autoantibodies against different aminoacyl transfer RNA (tRNA) synthetases. Morbidity and mortality of this disease are highly affected by interstitial lung disease (ILD) which is present in about 80% of patients. In this study, we investigated possible differences in 84 immune-related circulating miRNAs between ASSD patients with and without ILD; we enrolled 15 ASSD patients, 11 with ILD (ILD+) and 4 without ILD (ILD-), and 5 patients with idiopathic pulmonary fibrosis (IPF) as an additional control group. All patients were at disease onset and not on therapy at the time of inclusion. Differentially expressed miRNAs were identified in plasma-derived exosomes, using an miRNA PCR array (MIHS-111ZG, Qiagen, Hilden, Germany); miR-30a-5p and miR-29c-3p were upregulated in ASSD-ILD patients compared to patients without lung involvement (adjusted *p*-value < 0.05). IPF patients showed higher miR-29c-3p expression levels with respect to both ASSD and ASSD-ILD (*p* = 0.0005), whereas levels of miR-30a-5p were not different. miR-29c-3p and miR-30a-5p are overexpressed in ASSD-ILD+ patients compared with ILD−. These miRNAs are involved in the regulation of inflammation and fibrosis through their action on NF-κB and TGF-β1. Although the mechanistic role of these miRNAs in ASSD-ILD development has to be elucidated, we suggest that their exosome levels could be useful in identifying patients at risk of ILD.

## 1. Introduction

Antisynthetase syndrome (ASSD) is a systemic autoimmune disease referring to idiopathic inflammatory myopathies (IIMs) characterized by the positivity of specific autoantibodies addressed to various aminoacyl tRNA synthetases (ARS) [1], and by the occurrence of the classic clinical triad arthritis, myositis and interstitial lung disease (ILD) [2]. Of the different ARS, the most common is the anti-Jo1, reported in about 70% of cases, whereas the anti-EJ, anti-OJ, anti-PL7, anti-PL12, anti-KS, anti-Zo, and anti-YRS/Ha antibodies are less commonly detected [3]. Diagnosis is often challenging, due to the lack of established and shared classification criteria [4], and the occurrence of incomplete forms of the disease [5]. ILD is the most common and most prognosis-influencing manifestation of ASSD, reported in up to 80% of cases [3]. The treatment of ASSD-ILD relies on corticosteroids and immunosuppressants such as cyclosporine, tacrolimus, rituximab, and mycophenolate mofetil [6]. 

By considering the complexity and centrality of ILD in ASSD, the identification of biomarkers for lung involvement is crucial for the optimal follow-up of these patients and the possible identification of relevant involved pathways. Hence, in this study, we assessed for the first time, a panel of exosome-derived microRNAs, selected from the literature search because of their involvement in immune regulation, in a cohort of ASSD patients to analyze possible associations with the occurrence of ILD in these patients. Dysregulated miRNAs were also evaluated in a group of newly diagnosed patients with idiopathic pulmonary fibrosis (IPF) that has different pathogenic pathways.

## 2. Results

### 2.1. Demographic and Clinical Features of Patients

During the study period, we enrolled 15 ASSD patients: 11 with ILD and 4 without ILD. The clinical characteristics and ARS specificities of the included patients have been reported in Table 1. The median age of patients with ILD was 64 years (IQR 60–70 years) and of those without ILD, 62 years (IQR 52–71 years) (*p* = 0.948). All ASSD-ILD patients were females (100%), whereas two out of the four ASSD without ILD were males (50%) (*p* = 0.039). The most common ILD pattern we observed was non-specific interstitial pneumonia (NSIP) (91%), without (67%), or with (33%) areas of organizing pneumonia (OP).

### 2.2. miRNAs Expression Levels in Plasma-Derived Exosomes

We profiled the expression of 84 miRNAs (see Supplementary Table S1) involved in the activation and differentiation of T cells and B cells and potentially related to the pathogenesis of the disease. The data indicated that 59 of the 84 miRNAs (70%) were detectable (assay giving Ct values < 35 in at least one patient). Because no standard reference miRNA has been established for the normalization of exosome-derived miRNAs in plasma, we had to first determine the normalization references. Although the miScript PCR Array provides SNORD61, SNORD68, SNORD72, SNORD95, SNORD96A, and RNU6-6P as internal controls, which are often used for the normalization of cellular miRNAs, these internal controls were not useful for normalizing the levels of circulating miRNAs, since the levels of these miRNAs were very low or exhibited a high degree of sample-to-sample variation. To investigate the relative abundance of exosome miRNAs detected, the Ct values were, therefore, normalized using cel-miR-39 as an external spike-in control.

**Figure 1:**
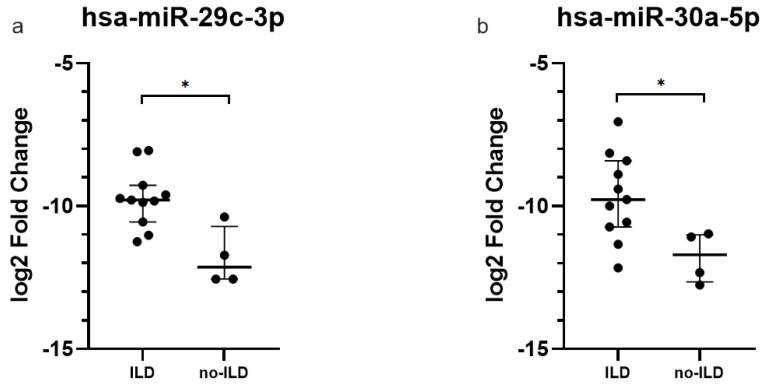
Quantitative expression of (**a**) hsa-miR-29c-3p, and (**b**) hsa-miR-30a-5p in the plasma-derived exosomes of ASSD patients with and without ILD. Log2 transformed values. *p*-value Bonferroni-corrected * < 0.05.

Only circulating levels of exosomal miR-29c-3p and miR-30a-5p were upregulated in ASSD-ILD patients compared to patients without lung involvement (Bonferroni adjusted *p*-value = 0.0358 and 0.0127, respectively, Figure 1). 

We reported the overall result of the 54 detectable miRNAs in Supplementary Table S2. 

**Figure 2:**
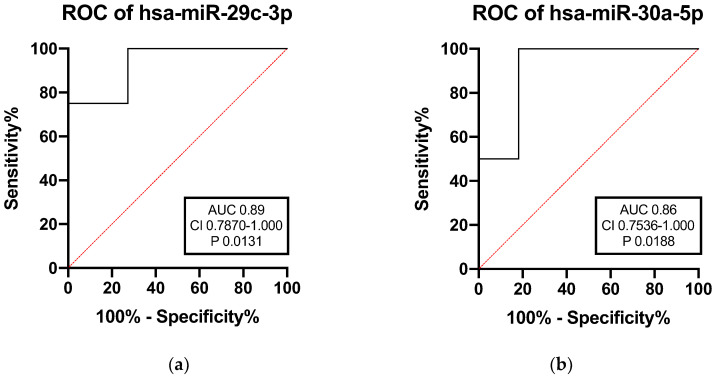
ROC curves analysis of circulating miR-29c-3p, (**a**) and miR-30a-5p, and (**b**) among ASSD patients with and without ILD. AUC: area under the curve. CI: confidence interval.

To evaluate the effectiveness of these miRNAs in predicting ILD in ASSD patients, ROC curve analysis was performed for the two upregulated miRNAs in ILD patients. The results indicated that levels of miR-29c-3p and miR-30a-5p discriminate ILD patients from patients without lung involvement with an area under the curve (AUC) of 0.89 and 0.86, respectively (Figure 2). 

### 2.3. Signaling Pathway Prediction and Targets Analyses

DIANA-miRPath analysis was applied to predict the biologic targets and pathways as well as cellular processes that miR-30a-5p and miR-29c-3p affected [7]. Eight KEGG biological processes were significantly enriched (*p* < 0.05) among dysregulated miRNAs from plasma exosomes in ILD patients (Table 2). 

### 2.4. Dysregulated miRNAs in IPF Patients

**Figure 3:**
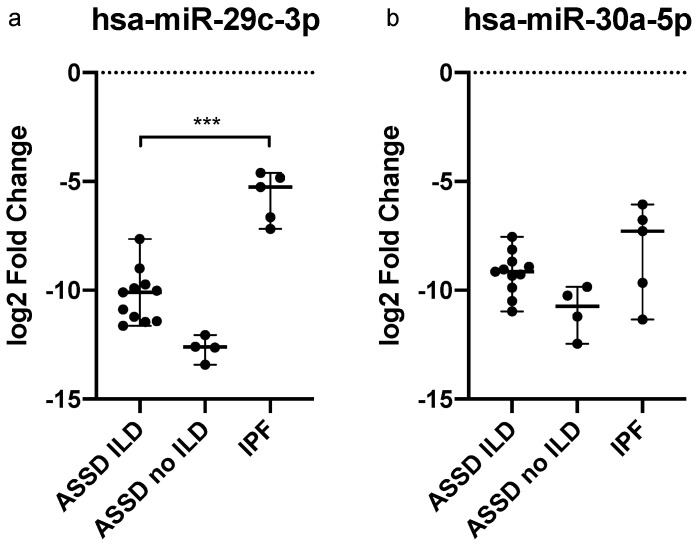
Quantitative expression of (**a**) hsa-miR-29c-3p, and (**b**) hsa-miR-30a-5p in the plasma-derived exosomes of ASSD patients (with and without ILD) and IPF patients. Log2 transformed values. *** *p* < 0.001.

The levels of deregulated miRNAs were then reassessed in the 11 patients with ASSD-ILD, 4 with ASSD no-ILD, and 5 patients de novo diagnosed with IPF. We included IPF patients in the study in order to focus our attention on the pulmonary involvement of ASSD patients and to possibly identify a correlation with miRNAs. IPF patients showed higher miR-29c-3p expression levels with respect to both ASSD no-ILD and ASSD-ILD (*p* = 0.0159 and *p* = 0.0005, respectively), whereas levels of miR-30a-5p displayed a great variability and did not differ significantly between ASSD (with or without ILD) and IPF (Figure 3). 

## 3. Discussion

In this proof-of-concept study, we showed that the levels of two exosome circulating miRNAs among the 84 that we had initially screened were associated with the occurrence of ILD in ASSD. MicroRNAs are short, noncoding, single-stranded RNAs that target complementary sequences of mRNA and finely regulate gene expression through post-transcriptional RNA silencing. MiRNAs control a wide variety of cellular processes and pathways such as cellular growth, proliferation, differentiation, regulation of the cell cycle, apoptosis, inflammation, and immune responses. Their dysregulation is involved in the development of a variety of autoimmune diseases, including dermatomyositis [8,9]. However, no evidence is available on the role of miRNAs in ASSD pathogenesis or clinical expression thus far. Furthermore, besides an inflammatory response, miR-30a-5p and miR-29c-3p also have a clear involvement in fibrogenesis. We identified miR-30a-5p and miR-29c-3p as significantly upregulated in ASSD with ILD compared to ASSD without ILD with a very good discrimination power according to ROC curves. MiRNAs were reported to play an important role in the regulation of the pathogenesis of IIM [10,11], however, being involved in numerous processes, the precise mechanisms in which they act are not entirely clear and will need to be better elucidated.

miR-29c has been proven to be a crucial regulator of B-cell maturation through direct interaction with RAG-1 (Recombination Activating 1) [12], and its expression is required for the survival of B cells. miR-29c is also implicated in the positive regulation of NK cells cytotoxicity [13], and, through the negative regulation of tumor necrosis factor alpha-induced protein 3 (TNFAIP3), it also modulates the activity of NF-kappa B in T cells [14]. Interestingly, miR-29c is upregulated in experimental models of sepsis [15] and ulcerative colitis [16], thus confirming its proinflammatory activity. Moreover, miR-29c seems to also display an antifibrotic action [17] through different mechanisms, such as the modulation of the Fer expression [18], and the blockade of the macrophage migration inhibitory factor [19]. Both the proinflammatory and the antifibrotic action of this miRNA might justify its upregulation in ASSD-ILD in the context of a high inflammatory response, and intense B cell activation, possibly associated with an attempt to limit the fibrotic evolution of the disease, but future, further mechanistic studies will be necessary to address its real biological significance. 

On the other hand, miR-30a-5p has been described as being involved in a greater number of biological processes, sometimes with conflicting results. It inhibits the proliferation and diffusion of many tumors, regulates the autophagy in chronic myelogenous leukemia, and reduces the epithelial-mesenchymal transition induced by TGF-β1 [20]. Furthermore, miR-30a-5p seems to play a double-edged role in inflammation, as shown in two experimental models [21,22]. The proinflammatory action is supported by the observation that miR-30a-5p was overexpressed in the noninfectious systemic inflammatory response syndrome, and correlated with the severity of the systemic inflammation [21]. Conversely, the anti-inflammatory action has been suggested in an experimental mouse model of acute lung injury, in which miR-30a-5p reduced the extent of Lipopolysaccharides (LPS)-induced damage through the increase in cell viability and cell cycle progression, the reduction in cell apoptosis, the influence on NF-Kb pathway, and the increase in IκBα degradation [22]. Moreover, miR-30a-5p could also display an antifibrotic action by reducing Smad2 levels in an experimental model of diabetic cardiomyopathy [23]. However, data on this matter are ambiguous. In fact, in an experimental model of viral myocarditis, the miR-30a-5p downregulation was linked to better cardiac outcomes, due to the reduction in the degree of myocardial fibrosis [24]. Zhang S et al., evaluated miR-30a expression in IPF patients, showing tha6 miR-30a could inhibit the Ten-Eleven Translocation 1 gene (TET1) [25], related to the pathophysiology of diffuse lung disease. Another study also showed that miR-30a-5p expression is downregulated in the bronchoalveolar lavage fluid in patients with IPF [26], which is not in line with our current data on plasma exosome levels. However, these results may indicate that miR-30a-5p can undergo organ-specific and disease-specific variations in its expression, possibly due to the local balance between proinflammatory and antifibrotic triggers. Thus, further studies are also necessary to unravel the exact biological activity of miR-30a-5p in the context of the pathogenesis of ASSD-ILD.

## 4. Materials and Methods

### 4.1. Study Population

Newly ASSD-diagnosed patients referring to participating centers (Pavia, Italy and Santander, Spain) from June 2019 to December 2020 were asked to participate in this cross-sectional and prospective study. For inclusion in the study, patients needed to have been free from immunosuppressive therapy (except for hydroxychloroquine up to 400 mg/day for no more than 2 months, and prednisone up to a maximum dose of 12.5 mg/day for no more than one month). All patients performed a full clinical and instrumental characterization that included the assessment of arthritis (clinical examination with joint count, joint X-rays, US assessment), myositis (creatine phosphokinase and aldolase testing, lower limbs muscle MRI, proximal electromyography), and ILD (pulmonary function tests and diffusing capacity of the lungs for carbon monoxide (DLCO), chest high resolution computed tomography). ASSD was defined as previously reported [2]. The Euroline autoimmune inflammatory myopathies 16 Ag kit (Euroimmun, Luebeck, Germany) was used in both centers for ARS determination. The patients were stratified according to ILD presence or absence. Before definitive inclusion, ASSD without ILD should have been followed for at least 12 months without the clinical and instrumental evidence of ILD occurrence. In all cases, peripheral blood samples were collected during the first local assessment. Blood samples were centrifuged at 2000 rpm for 15 min to separate plasma/serum and erythrocytes. Aliquots of plasma (at least two) were collected into RNase/DNase-free tubes and frozen at −80 °C until exosome isolation. The samples from Santander were shipped to Pavia in dry ice. Biological samples were then centrally evaluated for miRNA analysis. 

### 4.2. Exosomes and RNA Isolation

Plasma was centrifuged at 2000× *g* for 20 min to remove cells and debris and then the supernatant was centrifugated at 10,000× *g* for 20 min at room temperature to obtain the clarified plasma. Total exosomes were isolated from clarified plasma using a total exosome isolation kit (Invitrogen, Waltham, MA, USA), according to the manufacturer’s instructions. miRNeasy kit (Qiagen) was used to extract total RNA, following the manufacturer’s protocols. The concentration and purity of the total RNA were measured using a spectrophotometer (Nanodrop 2000, Thermo Scientific, Waltham, MA, USA).

### 4.3. Quantitative Real-Time Reverse Transcription PCR

Differentially expressed miRNAs in ASSD groups were identified using an miRNA PCR array (MIHS-111ZG. Qiagen) including 84 miRNAs involved in the activation and differentiation of T cells and B cells (see Supplementary Table S1). Firstly, reverse transcription for the total RNA sample was performed with the miScript II RT Kit (Qiagen) following the manufacturer’s protocol. Next, quantitative real-time PCR (qRT-PCR) was performed using LC480 Instruments (Roche, Basel, Switzerland) in 10 μL volume. Thermal cycling conditions consisted of initial denaturation at 95 °C for 15 min, followed by 45 cycles of 94 °C for 15 s, 55 °C for 30 s, and 70 °C for 30 s. 

The deregulated miRNAs were then reassessed in the ASSD and ASSD-ILD and evaluated in patients newly diagnosed with IPF by real-time PCR analysis using miRCURY LNA miRNA PCR-specific Detection Probe and miRCURY LNA SYBR Green PCR Kit (Qiagen).

### 4.4. miRNAs Target Prediction and Pathway Analysis

DIANA-miRPath v.2.0 was used to predict target genes and pathways common to deregulated miRNAs in the known KEGG (Kyoto Encyclopedia of Gene and Genome) pathway [7]. The graphical output of the program provides an overview of the parts of the pathways modulated by miRNAs. The statistical significance value associated with the identified signaling pathways and the biological process was calculated by the program.

### 4.5. Statistical Analysis

Continuous variables were presented as mean and standard deviation, or median and interquartile range (IQR); categorical variables were presented as frequency and proportions. Comparisons of continuous data were made using the Mann–Whitney U test for two independent samples of data, and with the Kruskal–Wallis test for multiple comparisons, followed by post hoc tests. When necessary, the Bonferroni correction was applied. Categorical data were analyzed using Fisher’s exact test. All Ct values obtained from RT-qPCR greater than 35 were considered below the detection levels of reaction. The fold-change for each miRNA was calculated as 2^−ΔCt^. Receiver–operator characteristic (ROC) curves and the area under the ROC curve (AUC) were used to assess the performance of the selected miRNAs to serve as diagnostic tools/biomarkers for detecting the early-stage ILD presence in ASSD patients. Statistical analyses were performed using GraphPad Prism (GraphPad Software, Inc., San Diego, CA, USA), All statistical tests were two-sided, and a *p*-value < 0.05 was considered statistically significant.

## 5. Conclusions

Data on plasma exosomal expression in IIMs are interesting but scanty and mainly addressed to dermatomyositis [8,9]. In this context, miRNA dysregulation seems to be potentially useful as a marker of ILD involvement in ASSD. Until now, information on circulating miR-29c-3p and miR-30a-5p has been completely lacking in both ASSD and IPF. Moreover, despite their action on inflammation and fibrosis being well documented in the literature, the results are sometimes conflicting and mostly pertain to clinical conditions not entirely similar to ASSD, such as IIMs or IPF.

It should be demonstrated if miR-29c-3p and miR-30a-5p are merely markers of ASSD-ILD or if they are involved in its pathogenesis. To this purpose, further studies are planned to define whether the production of the identified miRNAs originates from the lungs or peripheral immune cells. However, independently of the possible pathogenic role of these miRNAs that will be defined in the subsequent studies, with this current study, we first suggested that their peripheral exosomal expression could be useful in identifying those more prone to developing ILD.

## Figures and Tables

**Table 1:** Demographic and clinical data of study subjects.

Patients	Sex	Age	ARS	Myositis	Arthritis	ILD	Type of ILD
1	Female	76 years	Anti-Jo1	−	+	+	NSIP
2	Female	81 years	Anti-PL7	−	+	+	NSIP
3	Female	70 years	Anti-Jo1	+	+	+	NSIP + OP
4	Female	36 years	Anti-Jo1	+	+	+	NSIP
5	Male	47 years	Anti-Jo1	+	+	−	-
6	Male	54 years	Anti-PL7	+	−	−	-
7	Female	70 years	Anti-Jo1	−	+	+	NSIP + OP
8	Female	61 years	Anti-PL7	+	−	+	NSIP
9	Female	64 years	Anti-Jo1	+	−	+	NSIP
10	Female	61 years	Anti-Jo1	+	+	+	OP
11	Female	59 years	Anti-Jo1	−	−	+	NSIP + OP
12	Female	39 years	Anti-Jo1	−	−	+	NSIP
13	Female	66 years	Anti-Jo1	+	−	+	NSIP
14	Female	70 years	Anti-Jo1	+	+	−	-
15	Female	74 years	Anti-Jo1	+	+	−	-

**Table 2:** Biological pathways enriched by differentially expressed serum miRNAs in ASSD-ILD patients compared to no-ILD patients.

KEGG Pathway	*p*-Value	Genes
**Fatty acid biosynthesis**	<1 × 10^−5^	5
**Fatty acid metabolism**	1.39 × 10^−4^	11
**Viral carcinogenesis**	3.52 × 10^−3^	52
**Lysine degradation**	2.19 × 10^−3^	18
**Proteoglycans in cancer**	2.94 × 10^−2^	56
**Colorectal cancer**	2.18	25
**p53 signaling pathway**	6.48 × 10	25
**Hippo signaling pathway**	0.001118592	36

## Data Availability

Data related to this manuscript is contained within the article or Supplementary Material.

## Supplementary Materials

The supporting information can be downloaded at: https://www.mdpi.com/article/10.3390/ijms232314579/s1.
