# Supplementary material for: A Proof-of-Concept Analysis of Plasma-Derived Exosomal microRNAs in Interstitial Pulmonary Fibrosis Secondary to Antisynthetase Syndrome
Source: Int J Mol Sci. 2022 Nov 23;23(23):14579. doi: 10.3390/ijms232314579 (PMC9735694; doi:10.3390/ijms232314579)
Supplement: Supplementary file 1 [file ijms-23-14579-s001.zip › ijms-2007864-supplementary.pdf]

Table S1. List of miRNAs assessed in both ASSD patients groups.

hsa-let-7a-5p	hsa-miR-146b-5p	hsa-miR-19a-3p	hsa-miR-29b-3p
hsa-let-7b-5p	hsa-miR-147a	hsa-miR-19b-3p	hsa-miR-29c-3p
hsa-let-7c-5p	hsa-miR-148a-3p	hsa-miR-204-5p	hsa-miR-30a-5p
hsa-let-7d-5p	hsa-miR-150-5p	hsa-miR-20a-5p	hsa-miR-30b-5p
hsa-let-7e-5p	hsa-miR-155-5p	hsa-miR-20b-5p	hsa-miR-30c-5p
hsa-let-7f-5p	hsa-miR-15a-5p	hsa-miR-21-5p	hsa-miR-30d-5p
hsa-let-7g-5p	hsa-miR-15a-3p	hsa-miR-210-3p	hsa-miR-30e-5p
hsa-let-7i-5p	hsa-miR-15b-5p	hsa-miR-214-3p	hsa-miR-31-5p
hsa-miR-100-5p	hsa-miR-16-5p	hsa-miR-221-3p	hsa-miR-326
hsa-miR-101-3p	hsa-miR-17-5p	hsa-miR-222-3p	hsa-miR-331-3p
hsa-miR-106b-5p	hsa-miR-17-3p	hsa-miR-223-3p	hsa-miR-335-5p
hsa-miR-125b-5p	hsa-miR-181a-5p	hsa-miR-23a-3p	hsa-miR-342-3p
hsa-miR-126-3p	hsa-miR-181b-5p	hsa-miR-23b-3p	hsa-miR-346
hsa-miR-128-3p	hsa-miR-181c-5p	hsa-miR-24-3p	hsa-miR-34a-5p
hsa-miR-130b-3p	hsa-miR-181d-5p	hsa-miR-25-3p	hsa-miR-365b-3p
hsa-miR-132-3p	hsa-miR-182-5p	hsa-miR-26a-5p	hsa-miR-423-5p
hsa-miR-139-5p	hsa-miR-184	hsa-miR-26b-5p	hsa-miR-574-3p
hsa-miR-142-3p	hsa-miR-18a-5p	hsa-miR-27a-3p	hsa-miR-92a-3p
hsa-miR-142-5p	hsa-miR-191-5p	hsa-miR-27b-3p	hsa-miR-93-5p
hsa-miR-145-5p	hsa-miR-195-5p	hsa-miR-28-5p	hsa-miR-98-5p
hsa-miR-146a-5p	hsa-miR-199a-5p	hsa-miR-29a-3p	hsa-miR-99a-5p

Table S2. Median and Interquartile range of miRNAs analyzed in ASSD without and with ILD. P value Bonferroni corrected.

	ASSD no ILD	ASSD ILD	Adjusted P value
hsa-let-7a-5p	4.30 (4.92-3.437)	2.8 (4.878 - -0.6313)	NS
hsa-let-7b-5p	5.12 (5.895 - 3.875)	5.25 (6.4 - 4.351)	NS
hsa-let-7c-5p	7.05 (8.205 - 6.675)	7.235 (7.66 - 6.311)	NS
hsa-let-7d-5p	6.835 (7.36 - 6.415)	7.875 (7.93 - 7.32)	NS
hsa-let-7e-5p	6.685 (7.61 - 6.1)	6.185 (6.49 - 6.049)	NS
hsa-let-7f-5p	6.435 (7.585 - 5.5)	6.67 (7.21 - 5.985)	NS
hsa-let-7i-5p	10.64 (11.69 - 9.395)	10.8 (12.92 - 10.53)	NS
hsa-miR-100-5p	10.44 (11.42 - 8.324)	9.86 (12.63 - 9.52)	NS
hsa-miR-126-3p	7.44 (8.685 - 6.86)	8.743 (8.981 - 7.78)	NS
hsa-miR-139-5p	10.32 (11.87 - 9.095)	9.468 (9.9 - 9.035)	NS
hsa-miR-148a-3p	10.22 (11.31 - 9.65)	11.47 (12.31 - 10.64)	NS
hsa-miR-150-5p	6.53 (7.115 - 5.43)	6.858 (7.516 - 6.195)	NS
hsa-miR-15a-5p	12.97 (13.15 - 10.88)	18.03 (19.29 - 17.09)	NS
hsa-miR-15b-5p	7.015 (7.68 - 6.415)	8.618 (8.976 - 8.038)	NS
hsa-miR-16-5p	8.095 (9.1 - 6.28)	9.543 (11.21 - 8.154)	NS
hsa-miR-181b-5p	10.69 (11.99 - 9.088)	11.34 (14.38 - 10.5)	NS
hsa-miR-191-5p	8.565 (9.745 - 7.76)	12.4 (13.5 - 10.19)	NS
hsa-miR-195-5p	8.69 (9.595 - 7.265)	10.05 (11.85 - 9.76)	NS
hsa-miR-21-5p	3.55 (5.035 - 2.865)	5.205 (6.313 - 3.951)	NS
hsa-miR-223-3p	3.693 (5.024 - 2.593)	5.66 (6.293 - 3.891)	NS
hsa-miR-23a-3p	5.07 (5.425 - 3.905)	6.9 (7.08 - 5.996)	NS
hsa-miR-23b-3p	8.455 (10.87 - 7.85)	9.718 (10.33 - 9.226)	NS
hsa-miR-24-3p	7.605 (10.26 - 6.68)	8.49 (10.64 - 5.545)	NS
hsa-miR-25-3p	6.375 (7.08 - 5.51)	7.88 (8.001 - 7.29)	NS

hsa-miR-26a-5p	7.08 (8.165 - 5.89)	9.473 (10.88 - 8.349)	NS
hsa-miR-26b-5p	6.46 (7.565 - 5.87)	7.49 (7.73 - 6.675)	NS
hsa-miR-27a-3p	9.728 (11.01 - 8.316)	10.9 (12.01 - 9.78)	NS
hsa-miR-29a-3p	9.55 (10.57 - 8.715)	8.768 (11.15 - 8.21)	NS
hsa-miR-29c-3p	9.79 (10.56 - 9.27)	12.55 (13.17 - 11.93)	0.012669
hsa-miR-30a-5p	9.775 (10.73 - 8.415)	11.71 (12.65 - 11)	0.035762
hsa-miR-30c-5p	10.99 (12.53 - 9.14)	11.59 (12.15 - 10.52)	NS
hsa-miR-30d-5p	8.895 (10.11 - 7.675)	9.728 (10.85 - 7.879)	NS
hsa-miR-30e-5p	11.5 (12.89 - 10.43)	12.21 (13.03 - 10.62)	NS
hsa-miR-31-5p	11.66 (13.26 - 9.821)	10.47 (12.42 - 9.719)	NS
hsa-miR-326	11.35 (13.86 - 10.08)	12.44 (13.3 - 12.34)	NS
hsa-miR-342-3p	8.875 (9.53 - 8.395)	9.013 (10.54 - 8.694)	NS
hsa-miR-423-5p	5.103 (8.329 - 3.668)	8.2 (8.98 - 6.035)	NS
hsa-miR-574-3p	10.09 (10.55 - 8.605)	11.92 (13.8 - 10.04)	NS
hsa-miR-92a-3p	4.475 (5.815 - 3.93)	7.195 (9.028 - 5.704)	NS
hsa-let-7g-5p	10.07 (11.55 - 8.676)	10.05 (13.15 - 9.875)	NS
hsa-miR-106b-5p	13.44 (18.13 - 10.64)	12.09 (13.22 - 10.96)	NS
hsa-miR-145-5p	15.03 (18.37 - 12.61)	13.23 (15.94 - 11.49)	NS
hsa-miR-146b-5p	9.76 (14.49 - 9.073)	10.41 (11.33 – 8.67)	NS
hsa-miR-155-5p	16.65 (17.68 - 13.12)	14.24 (15.44 - 13.88)	NS
hsa-miR-15a-3p	14 (17.85 - 11.98)	11.68 (13.68 - 9.704)	NS
hsa-miR-181b-5p	10.69 (11.99 - 9.08)	12.52 (17.17 - 10.81)	NS
hsa-miR-19a-3p	11.79 (15.37 - 4.43)	14.17 (17.65 - 10.7)	NS
hsa-miR-19b-3p	11.84 (14.01 - 9.95)	11.22 (11.48 - 10.95)	NS
hsa-miR-204-5p	12.8 (15.37 - 11.54)	11.38 (13.85 - 10.06)	NS
hsa-miR-20a-5p	9.675 (13.39 - 5.185)	11.99 (12.23 - 11.75)	NS
hsa-miR-20b-5p	8.858 (17.26 - -22.14)	14.52 (17.38 - 14.11)	NS
hsa-miR-210-3p	12.29 (16.34 - 11.13)	12.15 (13.89 - 10.85)	NS
hsa-miR-27b-3p	11.81 (14.28 - 9.126)	12.03 (12.61 - 11.46)	NS
hsa-miR-30b-5p	12.38 (14.4 - 10.94)	12.12 (13.62 - 10.62)	NS
hsa-miR-331-3p	12.91 (15.24 - 12.49)	12.82 (15.02 - 11.04)	NS
hsa-miR-335-5p	11.26 (13.63 - 10.06)	11.98 (13.74 - 10.78)	NS
hsa-miR-346	12.18 (13.69 - 10.64)	11.99 (14.48 - 11.77)	NS
hsa-miR-34a-5p	13.55 (16.03 - 11.95)	13.5 (16.02 - 12.12)	NS
hsa-miR-93-5p	12.56 (14.95 - 11.63)	16.05 (14.52 - 11.58)	NS
